# Generation of aggregation prone N-terminally truncated amyloid β peptides by meprin β depends on the sequence specificity at the cleavage site

**DOI:** 10.1186/s13024-016-0084-5

**Published:** 2016-02-19

**Authors:** Caroline Schönherr, Jessica Bien, Simone Isbert, Rielana Wichert, Johannes Prox, Hermann Altmeppen, Sathish Kumar, Jochen Walter, Stefan F. Lichtenthaler, Sascha Weggen, Markus Glatzel, Christoph Becker-Pauly, Claus U. Pietrzik

**Affiliations:** Institute of Pathobiochemistry, University Medical Center of the Johannes Gutenberg University of Mainz, Duesbergweg 6, 55128 Mainz, Germany; Institute of Biochemistry, Unit for Degradomics of the Protease Web, Christian-Albrechts-University, Otto-Hahn-Platz 9, 24118 Kiel, Germany; Institute of Neuropathology, University Medical Center Hamburg-Eppendorf, 20246 Hamburg, Germany; Department of Neurology, Molecular Cell Biology, University of Bonn, 53127 Bonn, Germany; German Center for Neurodegenerative Diseases (DZNE) and Neuroproteomics, Klinikum rechts der Isar, Technische Universität München, 81675 Munich, Germany; Munich Cluster for Systems Neurology (SyNergy), Munich, Germany; Department of Neuropathology, Heinrich Heine University, 40225 Duesseldorf, Germany

**Keywords:** Alzheimer’s Disease, Meprin β, Metalloprotease, Amyloid precursor protein, Amyloid β, N-terminal truncation, APP mutations, Protein-protein interaction, Cell surface protein

## Abstract

**Background:**

The metalloprotease meprin β cleaves the Alzheimer’s Disease (AD) relevant amyloid precursor protein (APP) as a β-secretase reminiscent of BACE-1, however, predominantly generating N-terminally truncated Aβ2-x variants.

**Results:**

Herein, we observed increased endogenous sAPPα levels in the brains of meprin β knock-out (ko) mice compared to wild-type controls. We further analyzed the cellular interaction of APP and meprin β and found that cleavage of APP by meprin β occurs prior to endocytosis. The N-terminally truncated Aβ2-40 variant shows increased aggregation propensity compared to Aβ1-40 and acts even as a seed for Aβ1-40 aggregation. Additionally, we observed that different APP mutants affect the catalytic properties of meprin β and that, interestingly, meprin β is unable to generate N-terminally truncated Aβ peptides from Swedish mutant APP (APPswe).

**Conclusion:**

Concluding, we propose that meprin β may be involved in the generation of N-terminally truncated Aβ2-x peptides of APP, but acts independently from BACE-1.

**Electronic supplementary material:**

The online version of this article (doi:10.1186/s13024-016-0084-5) contains supplementary material, which is available to authorized users.

## Background

One of the major hallmarks of Alzheimer’s Disease (AD) is the accumulation of soluble and aggregated amyloid β (Aβ) peptides in the brains of AD patients. Aβ peptides are generated from the amyloid precursor protein (APP) in the amyloidogenic pathway through two consecutive cleavage events. Most prominent, BACE-1 (β-site APP cleaving enzyme 1) cleaves APP at the β-secretase cleavage site and generates the N-terminus of Aβ (described in [1]). As an aspartyl protease, BACE-1 is responsible for the majority of Aβ peptides generated in the acidic environment of endosomes and lysosomes [2–5]. Second, the γ-secretase complex cleaves the remaining 99 amino acids long C-terminal fragment (CTF/C99) and releases the C-terminus of Aβ. As both secretases are not restricted to a single site, Aβ peptides vary in length. BACE-1 can generate Aβ starting in position p1 or p11 (Aβ1-x/11-x) [3] whereas γ-secretase complex has several cleavage sites and can generate varying C-termini of Aβ [6–10]. Based on genetic evidence and BACE-1 knock-out studies it has been demonstrated that BACE-1 is the quantitatively most important enzyme to generate Aβ in mice overexpressing APP with the Swedish mutation (APPswe), which is associated with a distinct form of familial AD (FAD) [3, 11–15].

However, N-terminally truncated Aβ variants starting with the alanine in p2 (Aβ2-x), which cannot be attributed to BACE-1 activity, have also been described in AD patients [16–19]. We recently identified meprin β as a new enzyme in APP processing as it is able to generate Aβ peptides starting with aspartate in p1 and with alanine in p2 independent of BACE-1 activity [20, 21]. Meprin β, a type I transmembrane metalloprotease, has biological functions in inflammation and collagen assembly and is associated with diseases, such as inflammatory bowel disease and fibrosis (reviewed in [22, 23]).

Although BACE-1 acts as the major β-secretase in vivo generating most of the Aβ peptides at position 1, we suggest that meprin β may act as an alternative enzyme responsible for the release of small amounts of N-terminally truncated Aβ species.

**Fig. 1:**
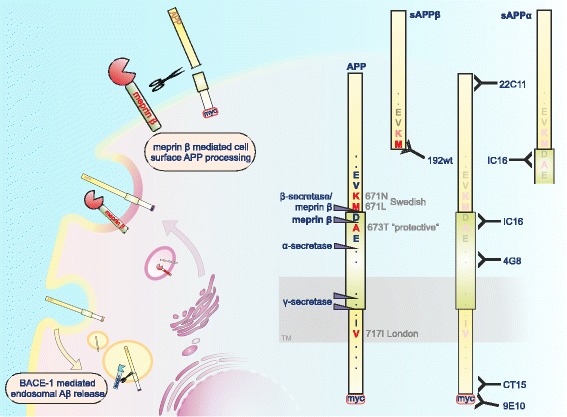
Schematic representation of the proteolytic cleavage of APP by meprin β. We found molecular interaction of APP and the metalloprotease meprin β in the secretory pathway and at the cell surface. On the right site, specific antibodies for APP/APP fragments and their epitopes are marked. Note that the neo-epitope 192wt antibody only recognizes “full-length” sAPPβ having methionine at the C-terminus, while the shorter sAPPβ starting with lysine produced via meprin β cleavage cannot be detected

The relevance of the protease for APP processing is further highlighted by its activity on endogenous APP in the mouse brain. Additionally we demonstrate that meprin β cleavage of APP occurs prior to the endocytic compartments, as diminished APP endocytosis has no influence on meprin β mediated Aβ generation (summarized in Fig. 1). Moreover, we are able to demonstrate that meprin β generates N-terminally truncated Aβ2-40/42 peptides that display increased aggregation compared to non-truncated Aβ peptides. This catalytic activity of meprin β is differentially affected by mutated forms of APP and in contrast to wt APP, meprin β is not able to cleave APPswe at position 672 and does not generate N-terminally truncated forms of Aβ from this APP mutant.

## Results

### A knock-out of meprin β leads to increased sAPPα secretion in cortical neurons

**Fig. 2:**
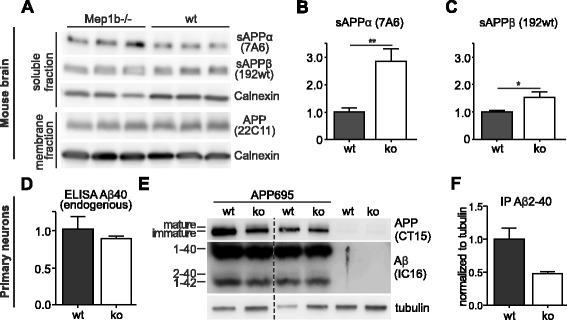
Increased sAPPα levels in meprin β ko mice. **a**-**c** Soluble and membrane fractions of brain lysates from meprin β ko (*Mep1b* ^*−/−*^) (*n* = 6) and wt (*n* = 6) mice were probed with antibodies specific for sAPPα (7A6), sAPPβ (192wt) (Additional file 1), full-length APP (22C11) and actin or tubulin as loading controls (note that only representative n = 3 for each is shown). **b** Note that endogenous sAPPα levels were increased in the absence of meprin β, indicating a changed APP processing profile in *Mep1b* ^*−/−*^ compared to wt mice. **d** Aβ40 levels of supernatants of primary cortical neurons of wt (*n* = 3) and meprin ko mice (*n* = 6) were detected via Meso Scale ELISA using sulfo-tagged 4G8 antibody. Meprin β ko mice showed approximately 13 % less Aβ40 (normalized to total protein of lysates). **e** Urea gel of immunoprecipitated Aβ (using IC16 antibody) of supernatants of primary cortical neurons of wt and meprin β ko mice infected with a recombinant adenovirus expressing APP695. Lysates showed a decrease in mature APP for meprin β ko (CT15 antibody). **f** Quantification of Aβ2-40 levels (normalized to tubulin of lysates) of (**e**) showed a decrease of approximately 50 % in meprin β ko neurons (*n* = 2)

We have previously shown that meprin β is able to cleave APP and to generate Aβ peptides in vitro [20, 21, 24]. We now wanted to elucidate the physiological role of meprin β in APP processing in vivo. Therefore, we analyzed endogenous APP processing in the brains of meprin β ko (*n* = 6) mice compared to age-matched wt animals (*n* = 6). Endogenous sAPPα and sAPPβ were detected in the soluble fractions and full-length APP was detected in membrane fractions of brain lysates (Additional file 1). Levels of endogenous full-length APP remained identical (Fig. 2a), but the release of endogenous sAPPα was increased in meprin β ko mice (Fig. 2a, b). This suggests that there might be more APP substrate for ADAMs in the absence of meprin β and supports the involvement of endogenous meprin β in APP processing in vivo (Additional file 2). The levels of sAPPβ were also slightly increased in meprin β ko mice (Fig. 2a, c), but note, that the sAPPβ specific antibody (192wt, Fig. 1) recognizes only the neo C-terminus of sAPPβ after BACE-1 (or minor meprin β) cleavage between M671/D672 and is therefore not able to detect the longer sAPPβ form generated by meprin β cleavage between D672 and A673. Overall, these findings demonstrate that meprin β affects endogenous APP processing in the mouse brain.

### Decrease of Aβ2-40 and increase of mature APP in primary neurons of meprin β knock-out mice

Since we detected differences in sAPPα levels for wt versus meprin β ko mice, we focused on total endogenous Aβ in supernatants of primary cortical neurons. Via ELISA assay we could show that total Aβ40 levels showed a trend to decrease (by about 13 %) in meprin β ko neurons (*n* = 6) compared to controls (*n* = 6) (Fig. 2d); however due to the antibody specificity we were unable to detect any endogenous mouse Aβ2-40. To circumvent this problem, we analysed N-terminally truncated Aβ variants and precipitated Aβ of supernatants of primary cortical neurons of the same mice infected with a recombinant adenovirus expressing APP695. We precipitated Aβ from cell supernatants 48 h post infection with subsequent separation on 8 M urea gels. We detected a decrease of Aβ2-40 levels in meprin β ko neurons compared to wt of about 50 % (Fig. 2e, f). Moreover, we observed an increase in levels of mature APP in neurons of meprin β ko mice compared to wt controls (Fig. 2e), indicating processing of fully glycosylated APP after posttranslational modification by meprin β.

### BACE-1 activity is not increased by meprin β preincubation

**Fig. 3:**
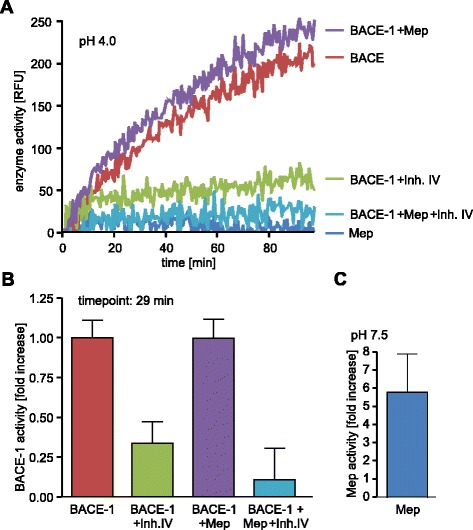
BACE-1 activity is not influenced by meprin β. **a**, **b** Proteolytic activity of recombinant BACE-1 or recombinant meprin β was measured at pH 4.0 using a quenched fluorogenic peptide comprising the β-site sequence of APP Swedish mutant. BACE-1 activity was inhibited using a specific inhibitor (Inh IV, Calbiochem). Preincubation of BACE-1, a mixture of 50 % mature and 50 % zymogen enzyme (R&D Systems), with meprin β revealed no additional increase in BACE-1 activity (**b**). **c** As control meprin β activity was additionally measured at pH 7.5

We have previously shown that generation of Aβ2-40 by meprin β in vitro is independent of BACE-1 [21]. However, since levels of sAPPα are increased in meprin β ko mice, we wanted to clarify if meprin β is capable of regulating BACE-1 activity directly. Close to the proposed activation site in the propeptide of BACE-1 a patch of negatively charged amino acids is found. Meprin β is known to prefer aspartate and glutamate residues around the cleavage site [24] and thus we performed a BACE-1 activity assay to test the potential role of meprin β acting as an activator of BACE-1 by cleaving off the propeptide (Fig. 3). We incubated C-terminally truncated soluble recombinant proBACE-1 with active meprin β and measured BACE-1 activity using a quenched fluorogenic peptide (mca-VNLDAE-dnp) comprising the sweAPP cleavage site. BACE-1 activity at acidic pH was not increased after meprin β preincubation, indicating that there is no activity stimulating effect mediated by meprin β (Fig. 3a, b). Meprin β activity at pH 7.5 was measured as control (Fig. 3c).

### Co-immunoprecipitation reveals interaction of APP and meprin β

**Fig. 4:**
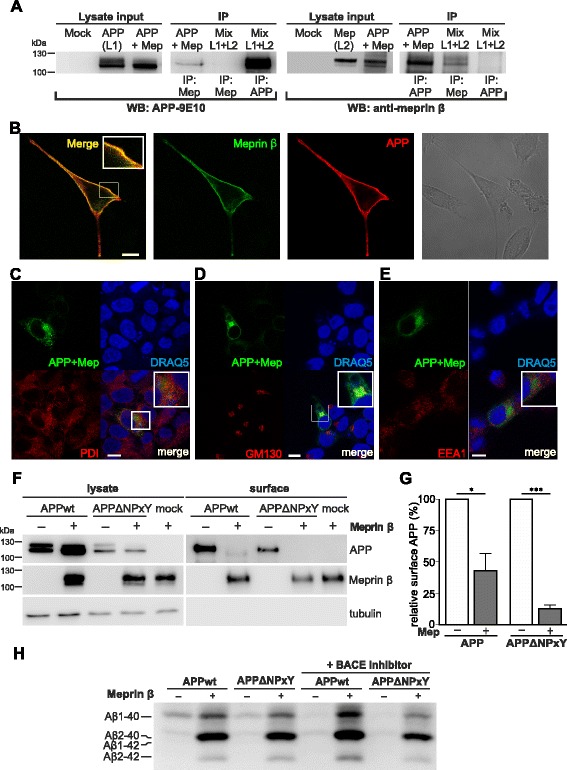
Cellular interaction between meprin β and APP occurs prior to endocytosis. **a** APP695myc and meprin β were overexpressed in HEK-293 T cells. Total lysates were immunoprecipitated with 9E10 (for APP) or anti-meprin β antibody. Co-purified proteins were detected using antibodies as indicated (WB: 9E10 for APP and reciprocal WB: meprin β). 20 μg of each lysate was used as transfection control (lysate input). As control, lysates of cells separately expressing either APP (“L1”) or meprin β (“L2”), were mixed post-lysis (lanes “Mix L1 + L2”). Co-purified proteins were immunoblotted as indicated. APP and meprin β specifically interacted only in co-transfected cells, but not post-lysis. **b** MEF cells were transiently co-transfected with APP695ΔNPxY and meprin β. Surface staining was performed, using IC16 antibody for detection of APP and an anti-meprin β antibody. Secondary antibodies Alexa-Fluor546 and Alexa-Fluor488 were used, respectively. The confocal image shows colocalization of APP (*red*) and meprin β (*green*). Single channels are depicted aside. The right image shows the cell morphology as bright-field picture (Scale bar: 10 nm). (C,D,E) GFP fluorescence shows colocalizing APP and meprin β (depicted in *green*) (Scale bar: 10 nm). **c** Only little colocalization of APP and meprin β can be found in the endoplasmic reticulum (PDI; depicted in *red*). **d** Colocalization was mostly found within the cis-golgi compartment (GM130; depicted in *red*). **e** Colocalization in early endosomes was hardly detectable (EEA1; depicted in *red*). **f**, **g** HEK-293 T cells were transiently co-transfected with APP695wt or APPΔNPxY, and meprin β or empty vector. Detection of proteins was carried out using specific antibodies. **f** 24 h post transfection, surface proteins were labeled using sulfo-NHS-LC-LC biotin and precipitated with NeutrAvidin agarose beads. **g** Quantification of biotinylated surface APP (graph shows mean ± S.E. (*n* = 3); statistical significance: * < 0.05, ***p < 0.001; *t*-test). **h** Aβ variants of conditioned medium was immunoprecipitated using IC16-conjugated dynabeads and separated on 8 M urea SDS-Page. Alternatively, cells were treated overnight with 100 nM tripartite BACE-1 inhibitor [26]. Samples were run on one gel, but rearranged for better presentation. Shown is one representative of six independent experiments. Generation of Aβ variants by meprin β was preserved in endocytosis impaired APPΔNPxY, indicating that cleavage occurred on the way to or at the cell surface

We have recently shown that APP is a substrate of the metalloprotease meprin β, even in BACE-1 knock-out cells [21]. However, in contrast to BACE-1 mediated cleavage of APP, nothing was known about the cellular localization of meprin β and APP interaction. To strengthen the involvement of meprin β in APP processing, we first explored whether APP and meprin β directly interact. We could show for the first time co-immunoprecipitation of myc-tagged APP and meprin β with the 9E10 antibody (anti-myc) from lysates of HEK-293 T cells transiently co-transfected with meprin β and APP695myc (Fig. 4a). We could clearly observe an effect of meprin β on APP processing in the lysate input control since levels of mature APP were decreased in co-transfected cells. Likewise, in a reverse experiment MEP1B antibody (anti-meprin β) co-precipitated transfected full length APP. Control experiments in which cells were single-transfected with APP (“L1”) or meprin β (“L2”) with subsequent mixing of the cellular lysates (Mix) confirmed that immunoprecipitation with 9E10 or the meprin β antibody only pulled either APP or meprin β showing that the interaction of APP and meprin β occurred only in the living cells and that APP and meprin β specifically interacted only in co-transfected cells.

### APP and meprin β co-localize in the late secretory pathway or at the cell membrane

To further analyse the cellular localization of the APP/meprin β interaction and meprin β enzymatic activity on APP processing, we used a well-established APP construct lacking the C-terminal motif NPxY (APPΔNPxY), which is critical for proper endocytosis [2]. Using this construct allowed us to investigate APP processing prior to the endocytic compartment, hence prior to the BACE-1 mediated Aβ generation. We performed a surface staining of MEF cells, transiently overexpressing meprin β and APPΔNPxY. To visualize only surface localized proteins we directly incubated the cells with anti-Aβ IC16 antibody [25] and anti-meprin β antibody after fixation and blocking without permeabilization to ensure that antibodies do not penetrate into the cell. The confocal images nicely show a co-localization of both proteins at the cell surface (Fig. 4b). As expected, our untransfected negative control and the controls of transfected cells that were only incubated with the corresponding secondary antibody, did not show any surface staining (data not shown).

To further highlight the colocalization of APP with meprin β, we co-transfected HEK cells with APP and meprin β constructs that are tagged with a fragment of the green fluorescent protein (GFP) (APP-GFP11, only bearing barrel 11 of GFP; meprin β-GFP1-10, only bearing barrel 1–10). Neither the GFP11 fragment nor the GFP1-10 fragment alone showed any fluorescence. However, when both fragments are in close proximity, the function of an intact GFP is regained and it is emitting fluorescence. Thus, fluorescence in cells co-transfected with APP-GFP11 and meprin β-GFP1-10 indicates a colocalization of both proteins. Of note, colocalization was mostly found in the secretory pathway within the cis-golgi compartment (GM130 antibody) (Fig. 4d), there was lesser colocalization within the ER (PDI) (Fig. 4c), however hardly any in early endosomes (EEA1) (Fig. 4e). We further validated the specificity of this proposed colocalization using immunocytochemical stainings with organelle markers of fixed HEK cells tranfected with APP-GFP and meprin β-dsRed (Additional file 3).

To elucidate a potential APP processing by meprin β at the cell surface, we transfected APP695wt or APPΔNPxY with or without meprin β into HEK-293 T cells and performed a cell surface biotinylation assay (Fig. 4f). The mature form of APP is cleaved by meprin β and is therefore reduced in cell lysates and at the cell surface. Surface APP695wt is almost completely cleaved and thus significantly reduced by 70 % when meprin β is also present at the cell surface (Fig. 4g). Surface APPΔNPxY is even stronger reduced by meprin β by 85 % when internalization is impaired. We further analyzed Aβ variants derived from these cells using 8 M urea SDS-Page. We could observe that an increased generation of Aβ1-40, Aβ2-40 and Aβ2-42 peptides by meprin β is not dependent on internalization. Furthermore, to prove a direct involvement of meprin β in Aβ generation we inhibited BACE-1 activity using different BACE-1 inhibitors like a BACE-1 tripartite inhibitor (described in [26]) or BACE-1 IV inhibitor (data not shown). Inhibition of BACE-1 activity did not alter the Aβ pattern generated by meprin β, but decreased levels of Aβ1-40 in cells lacking meprin β overexpression, which indicates the potency of the inhibitor (Fig. 4h). This suggests that meprin β cleaves APP and generates Aβ2-40/42 prior to the endocytic compartments as APPΔNPxY proteins remain at the cell surface and seem to serve as more accessible substrates for meprin β.

### Meprin β generated Aβ2-40 promotes and seeds aggregation of Aβ peptides

**Fig. 5:**
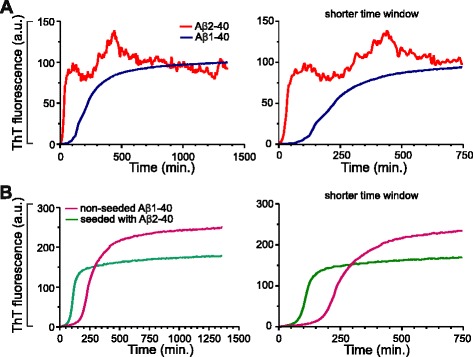
Aβ2-40 shows increased aggregation propensity and could seed the aggregation of non-truncated Aβ. Aggregation of synthetic Aβ peptide (truncated Aβ2-40 and non-truncated Aβ1-40) variants were monitored by Thioflavin T (ThT) fluorescence assay during incubation at 37 °C for 24 h (**a**). The aggregation assay was performed in 50 mM sodium phosphate buffer and a final Aβ concentration of 50 μM. The truncated Aβ2-40 peptide demonstrate a shorter lag phase and rapid aggregation behavior as compared to non-truncated Aβ1-40 variant. **b** Demonstration of the seeding capacity of Aβ2-40 peptides. Aggregation of Aβ1-40 with and without preformed oligomeric nuclei of Aβ2-40 was monitored by ThT fluorescence assay. Initial aggregate formation is accelerated by the addition of the Aβ2-40 seeds, as indicated by the reduced lag phase. The graphs on the right-side in (**a**) and (**b**) display the same results with extended time scale to demonstrate the differences in the lag phase. The differences are statistically significant. For Unpaired *t* test and F test, the *P* value is < 0.0001

As meprin β generates Aβ2-40 and Aβ2-42 and since N-terminally truncated Aβ variants can be found in the CSF and senile plaques of AD patients [16], we were interested in the aggregation of the truncated and non-truncated (wt) Aβ variants. Therefore, we compared the aggregation of truncated Aβ2-40 and the non-truncated Aβ1-40 variant. Aggregation was monitored by measuring the Thioflavin T (ThT) fluorescence upon binding to Aβ aggregates (Fig. 5a). Both truncated Aβ2-40 and non-truncated Aβ1-40 variants showed characteristic sigmoidal aggregation curves. Interestingly, truncated Aβ2-40 peptide showed significantly faster aggregation as compared to non-truncated Aβ1-40 peptide. The truncated Aβ2-40 peptide had a shorter lag phase of about ~12.5 min as compared to that of non-truncated Aβ1-40 of ~100 min. In addition, truncated Aβ2-40 also showed an increased elongation rate reaching a higher aggregation level than the Aβ1-40 peptide. A decay in ThT fluorescence was observed with Aβ2-40 peptide similar to the previously reported N-terminal truncated Aβ4-x peptide, probably due to the masking of ThT-binding sites within higher order aggregates or clumps [27]. These data indicate that the aggregation of N-terminally truncated Aβ2-40 peptide is significantly different from that of the non-truncated wt Aβ1-40 peptide. In particular, the Aβ2-40 species aggregate faster and reached a higher aggregation state than Aβ1-40 peptide.

We also investigated the seeding potency of the truncated Aβ2-40 variant. In a nucleation-dependent aggregation assay, we studied the effect of truncated Aβ variants on the aggregation of the non-truncated Aβ variant. Preformed oligomeric nuclei of truncated Aβ2-40 peptide significantly reduced the lag period of fibrillization (Fig. 5b). While Aβ1-40 alone showed a characteristic lag phase (~125 min.), preformed oligomeric nuclei of truncated Aβ2-40 strongly shortened the lag phase to ~60 min. Altogether, these data demonstrate that the truncated Aβ2-40 aggregates rapidly, and also could efficiently seed the aggregation of non-truncated (wt) Aβ variants.

### The “protective” APP A673T mutation is less prone to cleavage by meprin β

**Fig. 6:**
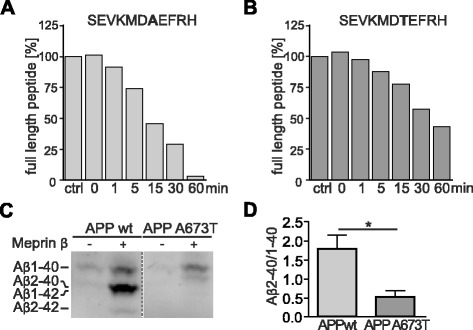
The “protective” APP A673T mutation decreases cleavage by meprin β. **a**, **b** 15 nM recombinant meprin β was incubated with synthetic APP peptides at 37 °C. HPLC analysis showed that processing kinetics of APP A673T were decreased (**b**) compared to wt APP (**a**) (see also Additional file 4). **c** Supernatants of HEK-293 T cells, transiently transfected with APPwt or APP A673T mutant and co-transfected with meprin β or empty vector were immunoprecipitated with anti-Aβ 6E10-Dynabeads, subsequently separated on an 8 M urea gel and probed with 6E10. The Aβ2-40 band, visible in samples transfected with APPwt and meprin β, is slightly shifted in samples transfected with APP A673T and meprin β. All samples were run on one gel but rearranged for better presentation. **d** A significant decrease of the Aβ2-40/1-40 ratio was observed in culture supernatants of cells co-transfected with APP A673T and meprin β compared to cells co-transfected with APPwt and meprin β (graph shows mean ± SEM (*n* = 5); statistical significance: *, *p* = 0.0317; *t*-test)

A recently described APP mutation in position 673 (A673T) has been shown to protect against AD as well as against cognitive decline in the elderly independently of AD [28–30]. This mutation is located adjacent to the β-secretase cleavage site in the Aβ sequence at p2 and reduces Aβ generation by 40 % in vitro. [28]. According to the findings reported above we speculated that this amino acid exchange (A673T) may also influence the affinity of meprin β towards APP. To investigate the influence of this mutation on meprin β cleavage of APP, we performed a cleavage assay using recombinant enzyme and synthetic peptides including the A673T mutation. HPLC and subsequent MALDI analysis revealed preferred cleavage of the wt over the A673T APP peptide by meprin β (Fig. 6a, b; Additional file 4). Indeed, meprin β prefers alanine over threonine in P1’ position [24], which may explain reduced cleavage of APP A673T by meprin β.

To further clarify Aβ generation by meprin β from APP A673T in vitro, we compared Aβ variants generated of APPwt or APP A673T by meprin β (Fig. 6c). In APP A673T transfected cells we observed no change in Aβ1-40 levels compared to those of APPwt. Furthermore, we detected a distinct signal below the Aβ1-40 band, which could not be attributed to one of the Aβ marker peptides. However, we suggest that this band is also representing Aβ2-40 (Additional file 5), but starting with a threonine at position two and shifted due to changes in hydrophobicity. Therefore, we compared the Aβ2-40/1-40 ratio of APP695 wt and A673T mutant co-transfected with meprin β (Fig. 6d). Our analysis revealed a significant decrease of ~70 % in the Aβ2-40/1-40 ratio in the A673T mutant due to reduced Aβ2-40 production (Fig. 6d).

### APPswe mutation affects meprin β cleavage

**Fig. 7:**
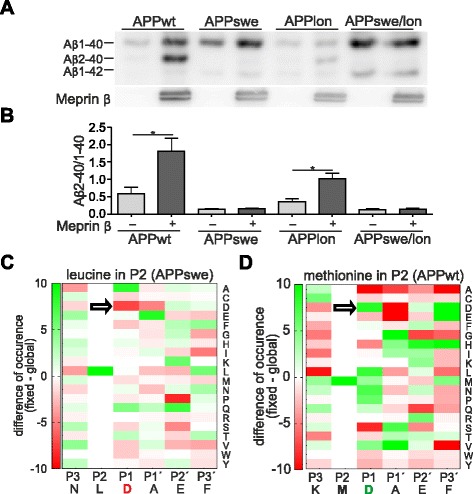
Meprin β cleavage is affected by mutations in APP. **a**, **b** Western Blots of 8 M urea gels showed a distinct Aβ2-40 band in HEK-293 T cells transiently co-transfected with APP695wt or APPlon and meprin β. Supernatants were immunoprecipitated for Aβ using 6E10 antibody. Note that APPswe or APPswe/lon transfected cells, co-transfected with meprin β, did not show the Aβ2-40 band (graph shows mean ± SEM (*n* = 4); statistical significance: * < 0.05; *t*-test). **c**, **d** Subsite cooperativity determines the meprin β cleavage site in APP. Previously identified meprin β cleavage sites in peptides derived from HEK-293 T cell lysates [24] were analyzed by a web-based tool [31] to visualize changes in the preference for certain amino acid residue around the scissile bond (*black line*). Green color indicate high, red color low preference. Aspartate in P1 position is well preferred in APPwt, but disliked in APPswe

We have recently shown that meprin β overexpression results in increased levels of Aβ2-40. Therefore, we decided to use the Aβ2-40/1-40 ratio as a measure for meprin β activity. In contrast to BACE-1, meprin β exhibits the same increased affinity for APPwt peptides and those carrying the Swedish mutation (K670N/M671L) in vitro [21]. However, here we demonstrate that generation of the N-terminally truncated Aβ2-40/42 variants by meprin β from APP carrying the Swedish FAD mutation (APPswe) was almost completely abolished compared to APPwt controls (Fig. 7a, b). To analyse whether other FAD mutations may affect meprin β activity in a similar fashion or whether only mutations localized closely to the N-terminus of the Aβ sequence influence meprin β cleavage specificity, we also investigated the FAD causing London APP mutation (APPlon, V717I). Using APPlon we observed that the ratio of Aβ generation in meprin β co-expressing cells is comparable to APPwt, indicating that changes close to the N-terminal sequence of the Aβ region may directly affect meprin β cleavage specificity. Additionally, we tested the Swedish/London double mutation and could not detect a signal for Aβ2-40 which can be therefore attributed to the APPswe mutation. To further support this, we employed data from a previously performed peptide cleavage assay [24], and analyzed with the help of a web-based tool [31], if the change of methionine (APPwt) to leucine (APPswe) alters the preference of meprin β for certain amino acid residues around the cleavage site (Fig. 7c, d). Indeed, while aspartate in P1 position (nomenclature by Schechter & Berger [32]) is highly preferred in APPwt, this residue is clearly disliked at this particular position when leucine is set in P2, explaining the loss of Aβ2-40/42 generation by meprin β in APPswe.

Overall, we could clearly show that the amino acid composition around the β-site in APP affect meprin β cleavage preference. This is most striking for APPswe, which completely abolished Aβ2-40 generation.

## Discussion

The proteolytic cleavage of the amyloid precursor protein and the accumulation of Aβ peptides in the brain are key events in the pathogenesis of AD [10, 33]. It was shown that the deletion of BACE-1 in transgenic mice overexpressing APPswe abolishes Aβ generation in the brain [15, 34–36] and rescues behavioral and electrophysiological deficits [34, 37]. However, several N-terminally truncated Aβ variants have also been described in the CSF and brains of AD patients and cannot be attributed to BACE-1 activity [1, 16–19, 38–40]. This suggests that proteases other than BACE-1 may be involved in the generation of N-terminally truncated Aβ peptides in the brain. Since we could already show that the metalloprotease meprin β generates N-terminally truncated Aβ peptides in vitro [21] we propose that it may be a candidate for this cleavage event. We already showed a different APP cleavage pattern in brains of meprin β ko compared to wt mice, suggesting that meprin β is involved in N-terminal processing of endogenous APP in vivo [20]. In this study, we observed increased levels of endogenous sAPPα in meprin β ko mice. sAPPα is generated through cleavage at the α-secretase cleavage site within the Aβ sequence by ADAM10 [41–46]. A similar increase in sAPPα levels has been observed in BACE-1 ko mice or after treatment of mice with BACE-1 inhibitors [35, 47, 48]. We speculate that due to the absence of meprin β more substrate is available for ADAM10 cleavage. Since the 192wt antibody does only recognize the shorter sAPPβ variant mainly produced by BACE-1cleavage, it does not reflect the total sAPPβ load (Fig. 1). Therefore, we can see an increase of this variant in meprin β ko mice, since more substrate is available for BACE-1. Additionally, we detected a decrease of approximately 13 % of endogenous Aβ40 levels in meprin β ko neurons compared to wt controls. To gain further insights into Aβ production of endogenous meprin β we infected primary meprin β ko and wild-type control neurons with recombinant adenovirus expressing human APP695. These showed a decrease of approximately 50 % of Aβ2-40 levels compared to wt neurons. The remaining Aβ2-40, which is also present in the ko, may be explained by other proteolytic events like N-terminal truncation of the full-length Aβ originally generated by BACE-1, through aminopeptidases like APA [49]. We further wanted to elucidate whether there are other possible explanations for an increase of β-site cleavage in meprin β wt versus ko mice, like an activation of BACE1 via meprin β. The propeptide of BACE-1 is cleaved off by furin or other proprotein convertases within the secretory pathway [50]. Interestingly, Creemers and colleagues showed that there is β-secretase activity towards APP which is not influenced by the presence of the propeptide. However, to exclude a potential role of meprin β as BACE-1 cleaving and thus activating enzyme, we performed an activity assay. Our results illustrate that meprin β has no effect on BACE-1 activity under pH conditions of active BACE-1.

As the interaction of BACE1 with APP at the cell surface and in early endosomes has been demonstrated [51, 52], we were therefore interested whether we could detect a direct interaction of the metalloprotease meprin β and its substrate APP. To further elucidate in which cellular compartment meprin β mediated Aβ generation occurs, we used an APP construct with impaired endocytosis due to a deletion of the tyrosine dependent sorting signal (NPxY motif; ΔNPxY) at the C-terminus [2, 53]. Since APP is endocytosed, most of the Aβ1-40/1-42 generation and BACE-1 activity is found in endosomes due to its acidic pH optimum as an aspartyl protease [3–5]. Meprin β, however, belongs to the astacin family of zinc endopeptidases and is mainly active at or near the cell surface as a membrane-bound enzyme. Recently we could show that a hyperactive mutant of meprin β, which was exclusively localized at the secretory pathway and not secreted, resulted in enhanced APP shedding and Aβ generation [54]. Additionally, rat meprin β was shown to exhibit an acidic pH optimum with additional proteolytic activity at basic pH [55]. Therefore, it is possible that meprin β cleaves APP in the late secretory pathway, at the cell surface or – like BACE-1 – in endosomes. To exclude the endosomal pathway for meprin β, we used APPΔNPxY, which allows to normal post translational modification, maturation and transport to the cell surface. However, internalization and degradation of APPΔNPxY is impaired and thus BACE-1 mediated Aβ generation is strongly reduced [2, 56, 57]. We analyzed the processing of full-length APP and the generation of N-terminally truncated Aβ in cells co-expressing APPΔNPxY and meprin β. Mature surface APP almost completely vanished through cleavage of meprin β and this effect was even greater when endocytosis of APP was impaired. In contrast to BACE-1, meprin β was still able to cleave APP and to generate N-terminally truncated Aβ peptides when internalization of APP is decreased. Thus, our results demonstrate a spatial segregation of APP cleavage by meprin β from BACE-1 mediated APP cleavage. We hypothesize that still most of the initial APP processing of the amyloidogenic pathway occurs in the endosomal compartment by BACE-1, but that the N-terminally truncated Aβ2-40 and Aβ2-42 variants are generated at or close to the cell surface by meprin β. Our immunofluorescence data using the split-GFP assay combined with antibody stainings against transfected proteins further support these results, since we could detect meprin β and APP/APP ΔNPxY in close proximity prior to internalization. Moreover, a recent study described that a hyperactive mutant of meprin β led to massive shedding of APP already within the secretory pathway, again pointing to a potential interaction of the two proteins before endocytosis [54].

Some studies suggest that N-terminally truncated Aβ peptides may act as seeds and promote the aggregation of Aβ1-40 peptides with detrimental effects. So far, many studies have examined the characteristics of Aβ3-x, which can be N-terminally cyclized to pyroglutamate Aβ (pEAβ3-x) [39, 58–61], Aβ4-x [1, 27, 62] and Aβ5-x [38, 40, 63]. We have found meprin β mediated generation of Aβ2-x and therefore analyzed its aggregation and nucleation propensities. Indeed, Aβ2-40 has a high tendency to form aggregates compared to Aβ1-40. Furthermore, we studied the effects of an Aβ variant with high aggregation propensity over a variant with only low and could show that Aβ2-40 peptide nucleates the aggregation of Aβ1-40 in a dose-dependent manner. To our knowledge, this is the first study characterizing Aβ2-40 showing that it has a high aggregation propensity and nucleates the aggregation of non-truncated Aβ1-40 peptides in vitro.

Since several mutations within the APP sequence have been shown to have an impact on BACE-1 cleavage affinity on APP, we analyzed a recently described APP mutation A673T that has been shown to protect against AD as well as against cognitive decline in the elderly independent of AD. The mutation lies at position two of Aβ (Aβ-A/T) and has been shown to reduce BACE-1 mediated Aβ generation by 40 % using synthetic peptides as substrates [28]. Moreover, a significantly decreased Aβ production in human APP A673T-overexpressing primary neurons has been observed [30, 64]. Additionally, a decreased aggregation propensity of Aβ-A/T could be measured, which is showing the complexity of the protective effects of the substitution. Thus, we analyzed meprin β mediated cleavage of APP A673T in cell culture and in a peptide cleavage assay and revealed a significant decrease of ~70 % in the Aβ2-40/1-40 ratio compared to wt APP. This is consistent with the data of our cleavage assay which focused on the cleavage preference of meprin β by using recombinant enzyme and synthetic peptides including the mutation and analysis via HPLC and subsequent MALDI. Here, we detected the preference of alanine over threonine in P1’ position [24]. Surprisingly, we observed that Aβ2-x variants were missing in cells overexpressing meprin β and APP bearing the Swedish double mutation K670N/M671L (APPswe) which is located in close vicinity of the β-secretase cleavage site. Here, we found a significantly reduced meprin β mediated Aβ2-40/42 generation. Although BACE-1 is clearly the most prominent enzyme responsible for the generation of Aβ1-40 and Aβ1-42 peptides from the APPwt or APPswe sequences, we propose, that meprin β may be responsible for generating small amounts of N-terminal truncated Aβ2-40 and Aβ2-42 peptides almost exclusively from the APPwt sequences. This clearly shows a significant influence of amino acid substitutions around the β-secretase cleavage site for meprin β mediated Aβ generation. We suggest that the amino acid substitution close to the meprin β cleavage site provides a mechanistic explanation for the differential generation of truncated Aβ species from APPwt versus APPswe constructs.

## Conclusion

Concluding, we observed increased endogenous sAPPα levels in the brains of meprin β knock-out (ko) mice compared to wild-type controls. Moreover, we could show an interaction of APP and meprin β by co-immunoprecipitation in overexpressing cells. Furthermore, we could demonstrate that, besides BACE-1, which is by far the most important secretase responsible for the generation of Aβ, meprin β could be a subsidiary candidate protease in generating N-terminally truncated, aggregation-prone Aβ2-x peptides that have been described in AD patients. We also observed that the β-secretase activity of meprin β depends on the amino acid composition around the cleavage site in APP as demonstrated for the APPswe sequence and the AD protective mutant A673T. In particular, meprin β was incapable of cleaving APPswe at position 672 and generating N-terminally truncated Aβ from this mutant substrate.

## Methods

### Animals

Mature congenic *Mep1β*^*−/−*^ mice on a C57Bl/6 background, as previously described [65], were maintained on a 12-h light–dark cycle, with food and water ad libitum. Control and *Mep1β*^*−/−*^ animals were anesthetized by sodium pentobarbital overdose and sacrificed by cervical dislocation. Entire brains were removed and sub-dissected into cerebellum, frontal cortex, temporal cortex, hippocampus and “the rest” of the brain prior to further analyses. All mice were kept under specific pathogen-free conditions.

### Mouse brain lysates

Meprin β ko (*n* = 6) and wt littermates (*n* = 6) were sacrificed by decapitation. Snap frozen hemispheres were homogenized in 1 ml DEA buffer (50 mM NaCl, 2 mM EDTA, 0.2 % diethylamine, protease inhibitors) and insoluble material was precipitated by centrifugation. DEA fraction was ultracentrifuged at 100.000 x *g* for 30 min. The resulting supernatant was retained as the soluble fraction and neutralized by addition of 10 % 0.5 M Tris/ HCl, pH 6.8. The DEA insoluble material was homogenized with 1 % Triton-X lysis buffer and cleared by centrifugation [66]. Brain lysates were separated by SDS-PAGE and subsequently probed using monoclonal antibody (mAb) 7A6 specific for sAPPα, polyclonal antibody 192 specific for sAPPβ, mAb 22C11 recognizing the APP ectodomain, and actin for loading control [44]. For co-immunoprecipitation, brains were homogenized in lysis buffer (20 mM TrisHCl (pH 7.5), 150 mM NaCl, 0.5 % Triton X-100, protease inhibitors) [67].

### Enzyme Linked Immunosorbent Assay

Samples were analysed by the Aβ Triplex Immunoassay from Meso Scale Discovery using the sulfo-tagged 4G8 antibody for mouse Aβ detection. Aβ40 concentration was calculated using the MSD Discovery Workbench Software.

### Cortical cultures and infection

Primary cortical neurons were obtained from prenatal (E15) mice. Dissociated neurons were seeded at a density of 63,000 cells/cm^2^ on polyornithin (Sigma) precoated culture dishes and maintained in Neurobasal/B27 media (Gibco) supplemented with Glutamax (Gibco). Cells were infected with a recombinant adenovirus expressing human APP695 at a concentration of 100 pfu/cell for 6 h in DIV1 as described [68].

### BACE-1 activity assay

1 μg of C-terminally truncated recombinant proBACE1 (R&D systems) was incubated with 15 nM recombinant active meprin β at pH 7.5. Afterwards, pH was changed to pH 4.0 and BACE-1 activity was measured using a quenched fluorogenic peptide (mca-VNLDAE-dnp) comprising the sweAPP cleavage site. As control, BACE-1 inhibitor IV (Calbiochem) was applied.

### Cell culture, transient transfection and cell lysis

HEK-293 T cells were maintained and passaged in Dulbecco’s Modified Eagle Serum (DMEM) supplemented with 5 % fetal calf serum (FCS) and 0.5 % sodium pyruvate in an incubator at 37 °C and 5 % CO_2_. Transient transfection of HEK-293 T cells was performed using calcium-phosphate or FuGENE®HD (Promega). For all transfections the vectors pLHCX or pLBCX were used. 24 h post transfection, 293 T cells were lysed in NP-40 lysis buffer (500 mM Tris, pH 7.4, 150 mM NaCl, 5 mM EDTA, 1 % (v/v) Nonidet P-40, 0.02 % (v/v) Sodium Azide), plus Complete Protease Inhibitor Cocktail (Roche) for 20 min at 4 °C on ice. Subsequently cell debris was pelleted by centrifugation at 20.000 x *g* for 20 min at 4 °C in a microcentrifuge. Protein content of cleared lysates was determined by BCA assay (Pierce Chemicals, Rockford, IL, USA).

### Co-immunoprecipitation of APP and meprin β

HEK-293 T cells were transiently co-transfected with meprin β-pLBCX and APP695myc-pLHCX, or with either meprin β or APP plus empty vector as control. 24 h post transfection, 293 T cells were lysed in NP-40 lysis buffer (500 mM Tris, pH 7.4, 150 mM NaCl, 5 mM EDTA, 1 % (v/v) Nonidet P-40, 0.02 % (v/v) Sodium Azide), plus Complete Protease Inhibitor Cocktail (Roche) for 20 min at 4 °C on ice. 20 μg of total cell lysates were used as input control. For co-immunoprecipitation, equal amounts of total lysates (200 μg) were incubated over night with 30 μl of protein G sepharose beads (50 % (v/v) slurry) (GE Healthcare), and either monoclonal 9E10 antibody for myc-tagged APP at 1:20 dilution, or polyclonal anti-meprin β antibody, MEP1B (R&D Systems) at 1:100 dilution. Beads were collected by low speed centrifugation and washed three times with NP-40 lysis buffer. Bound proteins were eluted from sepharose beads with 30 μl 2x SDS sample buffer at 95 °C.

### Split GFP cloning

The plasmid pcDNA3.1-APP695 CT split GFP11 was used as template to subclone the APP695 CT split GFP11 construct into a pLHCX backbone with a 5´HindIII and a 3´ClaI restriction site 31. We used the plasmid pcDNA3.1-APP695 CT split GFP11 as a template to subclone meprin β CT split GFP1 10 with a 5´MluI und 3`ClaI restriction site into pLHCX.

### Immunofluorescence microscopy

Murine embryonic fibroblasts (MEF) or HEK-293 T cells were seeded on cover slides precoated with polyornithin. For surface staining MEF cells were transiently co-transfected with meprin β-pLBCX and APP695ΔNPxYmyc-pLHCX. For compartment staining HEK-293 T cells were transiently co-transfected with pLHCX-APP695 CT split GFP11 and pLHCX-meprin β CT split GFP1-10. 24 h post transfection, cells were fixed with 4 % PFA for 10 min at room temperature and, except MEF cells for the purpose of surface staining, permeabilized in 0.5 % Triton X in PBS for 20 min . For surface staining cells were washed three times with TBS before and after blocking in 4 % BSA. Cells were incubated with IC16 antibody (1:300), recognizing residues 1–16 of the human Aβ sequence, and anti-meprin β antibody (1:300), MEP1B (R&D Systems), for 2 h at 37 °C. For compartment staining cells were incubated with anti-PDI (abcam, 1:500), anti-GM130 (BD Transduction Lab, 1:300) or anti EEA1 (Abcam, 1:300) for 2 h at 37 °C. Primary antibody was removed and cells were washed four times with TBS. Respective secondary Alexa-Fluor546 and Alexa-Fluor488 antibodies (Invitrogen) were applied in a 1:500 dilution in TBS for 1 h at room temperature in the dark. A secondary antibody control as well as a control with cells that were not transfected was performed. The coverslips were embedded in ProLong Gold antifade reagent (Invitrogen). Z-stack images were acquired using a LSM710, AxioObserver confocal laser scanning microscope, Plan-Apochromat 63x/1.40 Oil DIC M27, using ZEN 2008 software (Carl Zeiss). A representative layer of z-stack is presented in the results part.

### Immunoprecipitation of amyloid β

Supernatants were normalized according to protein content of cell lysates 24 h post transfection. Immunoprecipitation (IP) with magnetic dynabeads M-280 was performed as described [69]. In brief, 5-fold concentrated IP detergent buffer (50 mM HEPES pH 7.4, 150 mM NaCl, 0.5 % (v/v) Nonidet P-40 (NP40), 0.05 % (w/v) SDS and protease inhibitor cocktail (Roche Complete) was mixed with supernatant. Magnetic dynabeads M-280 containing sheep-anti-mouse-IgG attached to their surface were precoated with 6E10 antibody (Covance) or IC16 antibody [25] for murine samples, both recognizing residues 1–16 of the human Aβ sequence, according to manufacturer’s protocol and added to samples. After incubation for 15 h at 4 °C samples were washed three times with PBS, 0.1 % BSA and once with 10 mM Tris–HCl, pH 7.5. Separation from beads was performed through boiling samples at 95 °C in 25 μl sample buffer (0.36 M Bis-Tris, 0.16 M Bicine, 1 % (w/v) SDS, 15 % (w/v) sucrose and 0.0075 % (w/v) bromophenol blue).

### Aβ separation with 8 M Urea SDS-Gel and Western Blot

Separation of immunoprecipitated Aβ peptides was performed with 0.75 mm polyacrylamide 8 M urea SDS-gels as described [70]. To achieve a better separation of Aβ peptides varying in length of only one to few amino acids a final concentration of 0.3 M H_2_SO_4_ was used for separation gels. The peptides were transferred to an Immobilion-P PVDF membrane via semi-dry Western Blotting (Biorad) at 47 mA/gel for 45 min. Membranes were boiled for 3 min in PBS and afterwards blocked in 5 % nonfat-dry milk in TBST for 30 min. 6E10 antibody (Covance, 1:1000 in TBST) or IC16 antibody was used for overnight immunostaining. After washing in TBST the membrane was incubated in secondary anti-mouse antibody (1:1000 in TBST) for 1 h.

### Western Blotting, Quantification and statistical analysis

Samples were eluted in SDS sample buffer and boiled at 95 °C. Total lysates and immunoprecipitates were then separated on 4-12 % NuPage (Novex, Invitrogen) gradient gels and transferred onto nitrocellulose membranes or, for Aβ separation, on Immobilion-P PVDF membranes after activation in methanol. After incubation with appropriate primary and secondary antibodies, immunoreactive bands were visualized using an ECL enhanced chemiluminescence system (Millipore). Western blots were quantified by densitometry using ImageJ 1.44. All graphs and statistical analyses were prepared using GraphPad Prism 4 software (GraphPad, La Jolla, CA, USA).

### Biotinylation

HEK-293 T cells were either transiently co-transfected with human myc-tagged APP695wt-pLHCX or APPΔNPxY-pLHCX and meprin β-pLBXC or with the empty vector alone and grown on 10 cm dishes to 90 % confluency. Biotinylation was performed as previously described [71]. In brief, cell surface proteins were biotinylated with 0.5 mg/ml Sulfo-NHS-LC-LC-Biotin (Pierce) in ice-cold PBS at 4 °C for 40 min. Cells were washed four times with ice-cold PBS containing 50 mM NH_4_Cl to quench unconjugated biotin and lysed in NP-40 buffer. Equal amounts of proteins were incubated with NeutrAvidin agarose resin (Pierce) at 4 °C overnight. Biotinylated proteins were recovered by boiling in 2 x SDS sample buffer for 5 min and separated on 4-12 % NuPage.

### Thioflavin T Binding Assay

Synthetic human Aβ1-40 and Aβ2-40 peptides were procured from VCPBio Lab. Lyophilized peptides were solubilized in 10 mM NaOH to a final peptide concentration of 230 μM, sonicated for 5 min in water bath (Brandelin Sonopuls) and stored at −80 °C until further use. Thioflavin T (ThT) binding assay was performed as described previously [72]. Briefly, Aβ1-40 or Aβ2-40 stock solution (230 μM) was mixed with 50 mM of sodium phosphate buffer (pH 7.4, 50 mM NaCl, 20 μM ThT and 0.01 % sodium azide) to final Aβ concentration of 50 μM. Aggregation kinetic measurements were carried out using a Varian Cary Eclipse fluorescence spectrophotomer. Samples were incubated at 37 °C with stirring. The ThT fluorescence was measured every 5 min for 25 h using excitation and emission wavelengths of 446 nm and 482 nm, with a slit width of 5 nm respectively. The samples were analyzed in duplicates in each measurement. The values in the graph represents the means from 3 independent measurements (*n* = 6).

### In vitro seeding

For seeding experiments, the previously aggregated Aβ2-40 peptide solutions were sonicated for 5 min in a water bath and 5 % (in volume and peptide concentration) aliquots of them were added to the fresh Aβ1-40 sample just prior to incubation at the amyloidogenic condition. The seeded aggregation of Aβ1-40 was then followed by ThT fluorescence measurement as described above. The samples were analyzed in duplicates. The values in graph represents the means from 2 independent measurements (*n* = 4).

### HPLC analysis

Synthetic APP peptides (SEVKMDA_376_EFRH or SEVKMDT_376_EFRH respectively) were incubated with 15 nM recombinant human meprin β in 20 mM HEPES for different time intervals at 37 °C. Recombinant meprin β has been produced as previously described [73]. The reaction was stopped with 0.1 % TFA/H_2_O. Afterwards, reverse phase HPLC was performed using a gradient from 2 % acetonitrile/ 0.1 % TFA up to 60 % acetonitrile/ 0.1 % TFA in 38 min. All peak fractions were collected and analyzed via MALDI MS.

## Additional files

Additional file 1: Figure S1. Antibodies 192wt and 7a6 only detect sAPP in brain soluble fractions of wildtype mice. To test the specificity of the antibodies 7A6 and 192wt we compared brain soluble fractions of wildtype and APP ko mice. Here, we could show that by using the corresponding antibodies both sAPPα and sAPPβ are detectable in brain soluble fractions of wildtype, but are lacking in those of APP ko. Since APLP2 can also be detected in APP ko brain membrane fractions, we show that the knock-out is restricted to APP and does not involve other members of the APP family. (PDF 646 kb) Additional file 2: Figure S2. ADAM10 protein levels are not altered in Mep1b−/− animals. To exclude that the lack of meprin β has any effect on ADAM10 levels, which could affect sAPP levels, we analyzed lysates of whole brains from 16 week old Mep1b−/− and wt animals via SDS-PAGE. By using a C-terminal polyclonal ADAM10 antibody (pineda) we detected neither a difference on levels of mature ADAM10 nor on levels of the pro form proADAM10. Therefore, we suggest that, when meprin β is lacking, the increase of sAPPα levels is indeed a result of higher substrate availability for ADAM10 which is independent on a lacking ADAM10/meprin β interaction. (PDF 566 kb) Additional file 3: Figure S3. APP and meprin β colocalize in the secretory pathway and at the cell surface. HEK cells were co-transfected with APP-GFP and meprin β-dsRed. Both proteins predominantly colocalize in the cis-golgi compartment. Colocalization in early endosomes was hardly detectable. (PDF 890 kb) Additional file 4: Figure S4. Meprin β prefers cleavage of wt APP compared to APP A673T. 15 nM recombinant meprin β was incubated with synthetic APP peptides for different time intervals. HPLC analysis showed that processing kinetics of APP A376T (F-J) were decreased compared to wt APP (A-E). Occurring peaks with smaller retention times were identified as cleavage products by MALDI MS. Peak intensity has been measured at 214 nm. (PDF 1715 kb) Additional file 5: Figure S5. APP A673T mutation protects from meprin β mediated cleavage. HEK-293 T cells were transiently transfected with APPwt or APP A673T mutant and co-transfected with the empty vector or meprin β. 24 h post transfection supernatants were immunoprecipitated using Dynabeads conjugated with a 6E10 anti-Aβ antibody, subsequently separated on an 8 M urea gel and probed with 1E8 anti-Aβ1-x/2-x (A). The blot was reprobed with 6E10 anti-Aβ antibody. All samples were run on one gel but rearranged for better presentation. (B) We could exclude a phosphorylation at the substituted threonine that causes a shift of Aβ2-40 by dephosphorylating with λ-phosphatase (New England Biolabs) after the last washing step of the immunoprecipitation. Samples were separated on an 8 M urea gel and probed with 6E10 anti-Aβ antibody that showed no change with or without phosphatase treatment (C). (PDF 936 kb)

## Abbreviations

AD: Alzheimer’s Disease
APP: Amyloid precursor protein
APPswe: Swedish mutant APP
Aβ: Amyloid β
CTF: C-terminal fragment
FAD: Familial AD
