# Supplementary material for: Generation of aggregation prone N-terminally truncated amyloid β peptides by meprin β depends on the sequence specificity at the cleavage site
Source: Mol Neurodegener. 2016 Feb 19;11:19. doi: 10.1186/s13024-016-0084-5 (PMC4759862; doi:10.1186/s13024-016-0084-5)
Supplement: Additional file 1: Figure S1. — Antibodies 192wt and 7a6 only detect sAPP in brain soluble fractions of wildtype mice. To test the specificity of the antibodies 7A6 and 192wt we compared brain soluble fractions of wildtype and APP ko mice. Here, we could show that by using the corresponding antibodies both sAPPα and sAPPβ are detectable in brain soluble fractions of wildtype, but are lacking in those of APP ko. Since APLP2 can also be detected in APP ko brain membrane fractions, we show that the knock-out is restricted to APP and does not involve other members of the APP family. (PDF 646 kb) [file 13024_2016_84_MOESM1_ESM.pdf]

Fig. S1

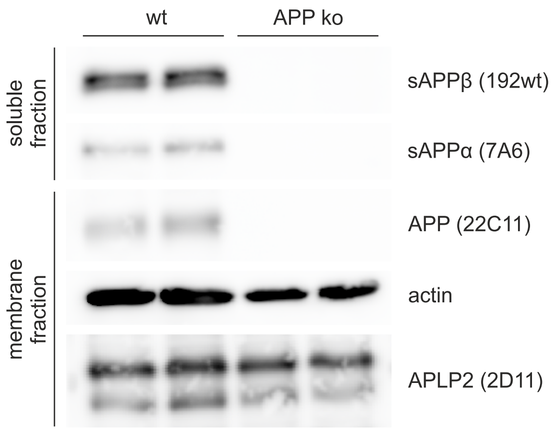

Additional Fig. 1: Antibodies 192wt and 7a6 only detect sAPP in brain soluble fractions of wildtype mice.

To test the specificity of the antibodies 7A6 and 192wt we compared brain soluble fractions of wildtype and APP ko mice. Here, we could show that by using the corresponding antibodies both sAPPα and sAPPβ are detectable in brain soluble fractions of wildtype, but are lacking in those of APP ko. Since APLP2 can also be detected in APP ko brain membrane fractions, we show that the knock-out is restricted to APP and does not involve other members of the APP family.
