# Supplementary material for: Generation of aggregation prone N-terminally truncated amyloid β peptides by meprin β depends on the sequence specificity at the cleavage site
Source: Mol Neurodegener. 2016 Feb 19;11:19. doi: 10.1186/s13024-016-0084-5 (PMC4759862; doi:10.1186/s13024-016-0084-5)
Supplement: Additional file 2: Figure S2. — ADAM10 protein levels are not altered in Mep1b−/− animals. To exclude that the lack of meprin β has any effect on ADAM10 levels, which could affect sAPP levels, we analyzed lysates of whole brains from 16 week old Mep1b−/− and wt animals via SDS-PAGE. By using a C-terminal polyclonal ADAM10 antibody (pineda) we detected neither a difference on levels of mature ADAM10 nor on levels of the pro form proADAM10. Therefore, we suggest that, when meprin β is lacking, the increase of sAPPα levels is indeed a result of higher substrate availability for ADAM10 which is independent on a lacking ADAM10/meprin β interaction. (PDF 566 kb) [file 13024_2016_84_MOESM2_ESM.pdf]

Fig. S2

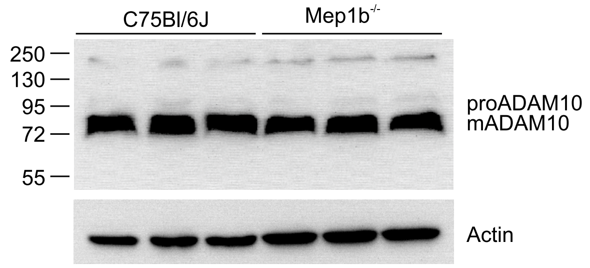

Additional Fig. 2: ADAM10 protein levels are not altered in Mep1b-/- animals.

To exclude that the lack of meprin β has any effect on ADAM10 levels, which could affect sAPP levels, we analyzed lysates of whole brains from 16 week old Mep1b$^{-/-}$ and wt animals via SDS-PAGE. By using a C-terminal polyclonal ADAM10 antibody (pineda) we detected neither a difference on levels of mature ADAM10 nor on levels of the pro form proADAM10. Therefore, we suggest that, when meprin β is lacking, the increase of sAPPα levels is indeed a result of higher substrate availability for ADAM10 which is independent on a lacking ADAM10/meprin β interaction.
