# Supplementary material for: Generation of aggregation prone N-terminally truncated amyloid β peptides by meprin β depends on the sequence specificity at the cleavage site
Source: Mol Neurodegener. 2016 Feb 19;11:19. doi: 10.1186/s13024-016-0084-5 (PMC4759862; doi:10.1186/s13024-016-0084-5)
Supplement: Additional file 3: Figure S3. — APP and meprin β colocalize in the secretory pathway and at the cell surface. HEK cells were co-transfected with APP-GFP and meprin β-dsRed. Both proteins predominantly colocalize in the cis-golgi compartment. Colocalization in early endosomes was hardly detectable. (PDF 890 kb) [file 13024_2016_84_MOESM3_ESM.pdf]

Fig. S3

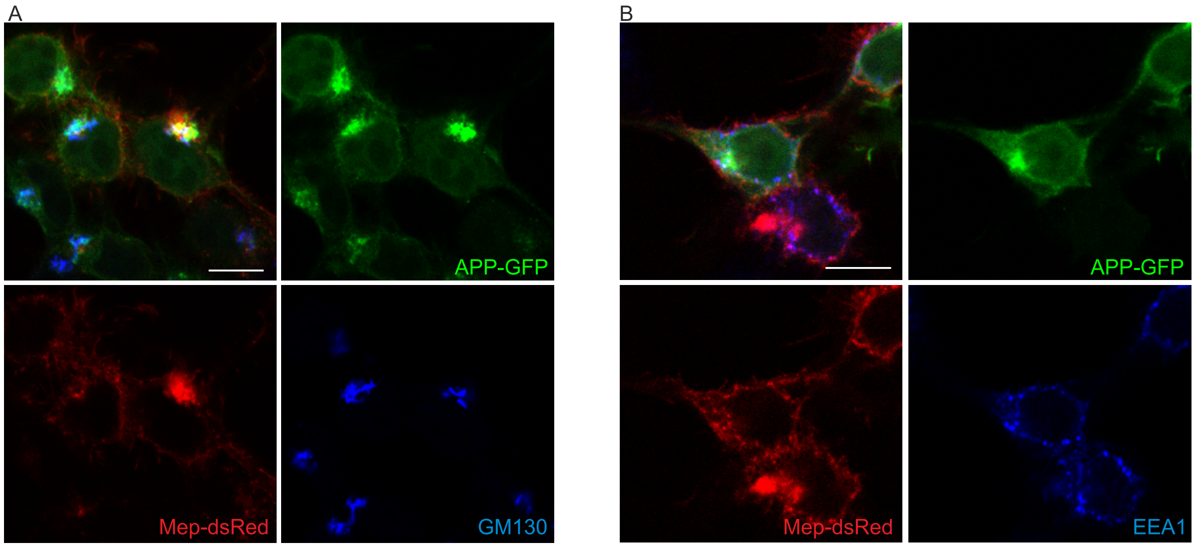

Additional Fig. S3: APP and meprin β colocalize in the secretory pathway and at the cell surface.

HEK293T cells were co-transfected with APP-GFP and meprin β-dsRed. Both proteins predominantly colocalize in the cis-golgi compartment. Colocalization in early endosomes was hardly detectable.
