# Supplementary material for: Generation of aggregation prone N-terminally truncated amyloid β peptides by meprin β depends on the sequence specificity at the cleavage site
Source: Mol Neurodegener. 2016 Feb 19;11:19. doi: 10.1186/s13024-016-0084-5 (PMC4759862; doi:10.1186/s13024-016-0084-5)
Supplement: Additional file 4: Figure S4. — Meprin β prefers cleavage of wt APP compared to APP A673T. 15 nM recombinant meprin β was incubated with synthetic APP peptides for different time intervals. HPLC analysis showed that processing kinetics of APP A376T (F-J) were decreased compared to wt APP (A-E). Occurring peaks with smaller retention times were identified as cleavage products by MALDI MS. Peak intensity has been measured at 214 nm. (PDF 1715 kb) [file 13024_2016_84_MOESM4_ESM.pdf]

Fig. S4

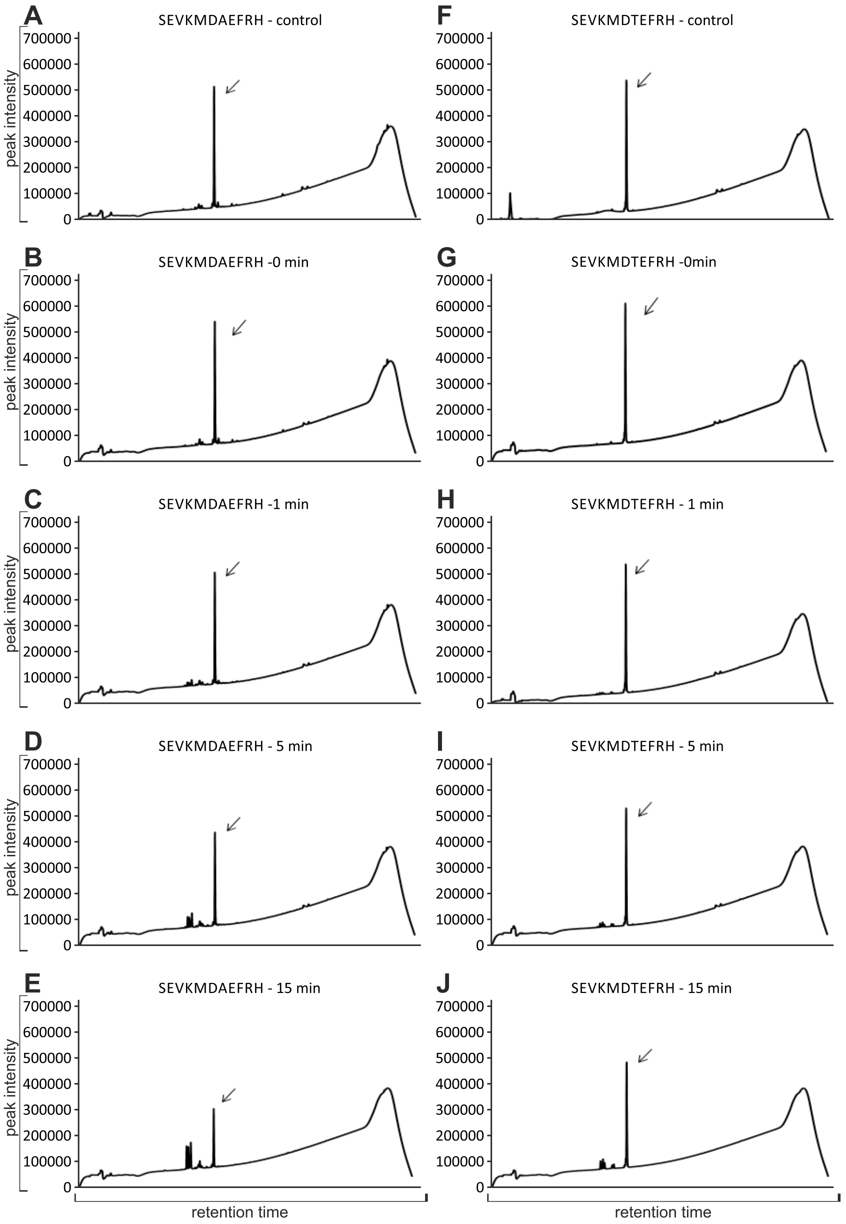

Additional Fig. S4: Meprin β prefers cleavage of wt APP compared to APP A673T. 15 nm recombinant meprin β was incubated with synthetic APP peptides for different time intervals. HPLC analysis showed that processing kinetics of APP A376T (F-J) were decreased compared to wt APP (A-E). Occurring peaks with smaller retention times were identified as cleavage products by MALDI MS. Peak intensity has been measured at 214
