# Supplementary material for: Generation of aggregation prone N-terminally truncated amyloid β peptides by meprin β depends on the sequence specificity at the cleavage site
Source: Mol Neurodegener. 2016 Feb 19;11:19. doi: 10.1186/s13024-016-0084-5 (PMC4759862; doi:10.1186/s13024-016-0084-5)
Supplement: Additional file 5: Figure S5. — APP A673T mutation protects from meprin β mediated cleavage. HEK-293 T cells were transiently transfected with APPwt or APP A673T mutant and co-transfected with the empty vector or meprin β. 24 h post transfection supernatants were immunoprecipitated using Dynabeads conjugated with a 6E10 anti-Aβ antibody, subsequently separated on an 8 M urea gel and probed with 1E8 anti-Aβ1-x/2-x (A). The blot was reprobed with 6E10 anti-Aβ antibody. All samples were run on one gel but rearranged for better presentation. (B) We could exclude a phosphorylation at the substituted threonine that causes a shift of Aβ2-40 by dephosphorylating with λ-phosphatase (New England Biolabs) after the last washing step of the immunoprecipitation. Samples were separated on an 8 M urea gel and probed with 6E10 anti-Aβ antibody that showed no change with or without phosphatase treatment (C). (PDF 936 kb) [file 13024_2016_84_MOESM5_ESM.pdf]

Fig. S5

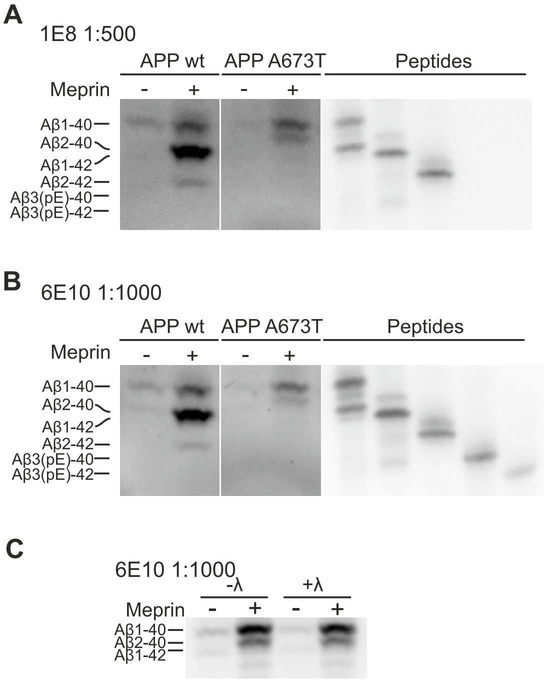

Additinal Fig. S5: APP A673T mutation protects from meprin β mediated cleavage. HEK-293T cells were transiently transfected with APPwt or APP A673T mutant and co-transfected with the empty vector or meprin β. 24 h post transfection supernatants were immunoprecipitated using Dynabeads conjugated with a 6E10 anti-Aβ antibody, subsequently separated on an 8 M urea gel and probed with 1E8 anti-Aβ1-x/2-x (A). The blot was reprobed with 6E10 anti-Aβ antibody. All samples were run on one gel but rearranged for better presentation. (B). We could exclude a phosphorylation at the substituted threonine that causes a shift of Aβ2-40 by dephosphorylating with λ-phosphatase (New England Biolabs) after the last washing step of the immunoprecipitation. Samples were separated on an 8 M urea gel and probed with 6E10 anti-Aβ antibody that showed no change with or without phosphatase treatment (C).
